# Editorial: ADAM, ADAMTS and Astacin Proteases: Challenges and Breakthroughs in the -Omics Era

**DOI:** 10.3389/fmolb.2021.780242

**Published:** 2021-10-12

**Authors:** Kazuhiro Yamamoto, Rens de Groot, Simone Dario Scilabra, Hang Fai Kwok, Salvatore Santamaria

**Affiliations:** ^1^ Institute of Life Course and Medical Sciences, University of Liverpool, Liverpool, United Kingdom; ^2^ Institute of Cardiovascular Science, University College London, London, United Kingdom; ^3^ Proteomics Group of Fondazione Ri.MED, Department of Research IRCCS ISMETT, Palermo, Italy; ^4^ Cancer Centre, Faculty of Health Sciences, University of Macau, Macau, China; ^5^ MOE Frontiers Science Center for Precision Oncology, University of Macau, Macau, China; ^6^ Department of Immunology and Inflammation, Imperial College London, London, United Kingdom

**Keywords:** ADAM, ADAMTS, proteomics, extracellar matrix, astacin

Metzincins are a superfamily of metalloproteinases characterized by the presence of a conserved methionine residue downstream the active site zinc (Cerdà-Costa and Gomis-Rüth, 2014). This Frontiers Research Topic is focused on three metzincin sub-families: ADAMs (A Disintegrin and Metalloproteinases), ADAMTS (A Disintegrin and Metalloproteinase with Thrombospondin motifs) and Astacins. Research into these proteases has seen impressive breakthroughs thanks to the progress of the -Omics era. Genome Wide Association Studies (GWAS) have implicated several ADAM and ADAMTS family members in human disease, for example ADAMTS7 in Coronary Artery Disease (Reilly et al., 2011), and ADAM10 in Alzheimer’s disease (Marioni et al., 2018; Schwartzentruber et al., 2021). Although less characterized, the proteolytic activity of Astacins has been linked to digestion, cytokine activation and cancer (Sterchi et al., 2008; Peters and Becker-Pauly, 2019).

ADAMTSs are a family of 19 secreted proteases playing a crucial role in the turnover of the extracellular matrix (ECM). While a fine regulation of their activity ensures tissue homeostasis, several pathological conditions are associated with their aberrant activity. Rose et al. comprehensively review the current knowledge about regulatory mechanisms of ADAMTS activity. Protein levels and activity of ADAMTSs can be modulated at different levels, including transcriptional regulation, alternative splicing, proteolytic activation, endocytosis, and inhibition by tissue inhibitors of metalloproteinases (TIMPs). A deeper understanding of such regulatory mechanisms can lead to new strategies to target the pathological potential of specific ADAMTSs without affecting the physiological activity of other family members. The contribution by Jiang et al. provides updated insights on multiple layers of regulation of one of the best characterized ADAMTS family members, ADAMTS5. ADAMTS5 is an important pharmaceutical target in osteoarthritis (OA) due to its ability to cleave aggrecan, the major proteoglycan in articular cartilage (Santamaria, 2020). Since no disease-modifying OA therapies are currently available, the authors summarize current preclinical approaches to target ADAMTS5. Monoclonal antibodies and small molecule inhibitors against ADAMTS5 were proved to be beneficial pre-clinically. Based on the recent novel RNA therapies that demonstrated potential in OA animal models, the authors emphasize that upstream signaling blockade of ADAMTS5 using mi/siRNAs may represent a feasible therapeutic strategy for OA.

ADAMTS12 is another example of how we are just starting to scratch the surface of ADAMTS complexity. Mohamedi et al. overview this complex and multifunctional metalloproteinase on the 20th anniversary of its identification. The authors discuss functions of ADAMTS12 in inflammatory processes, arthritis, degenerative intervertebral disc, chondrogenesis, tendon degeneration, cancer, neurological and fertility disorders. Different domains of ADAMTS12 can interact with different ECM components, which in turn modify its proteolytic activity. The authors emphasize that identification of ADAMTS12-binding partners would contribute to a deeper understanding of its biological functions.

A central aspect of this Research Topic is the identification of novel metzincin substrates using state-of-the-art proteomics approach. In the last decade, mass spectrometry-based techniques have dramatically expanded our knowledge of proteases’ substrate repertoire, the degradome (López-Otín and Overall, 2002). The most successful techniques have been those targeting the newly formed N-terminus generated following cleavage of the substrate, such as N-Terminal Amine Isotopic Labeling of Substrates (TAILS) (Mintoo et al., 2021). N-TAILS has been instrumental to elucidate the degradome of ADAMTS7 (Colige et al., 2019), 2, 3, and 14 (Bekhouche et al., 2016). While these approaches used fibroblast conditioned media as a source of substrates, Leduc et al. investigated substrates *in vivo* by directly comparing skin from *Adamt2* ^*−/−*^, *Adamts14* ^*−/−*^ and *Adamts2* ^*−/−*^/*Adamts14* ^*−/−*^ knockout mice with those of wild type littermates. Their results confirm the cleavage site preference (P1 and P1′ residues) of these proteases and provide more detail about their role in collagen fibril organization. As often in proteomics, further studies are required to assess the relevance of these intriguing proteolytic events and novel substrates.

The current pandemic has stressed the need for better intervention strategies aiming at the prevention and treatment of infections caused by respiratory viruses. As highlighted by Pedrina and Stambas, the ECM offers novel opportunities for intervention. The proteoglycan versican is a major ECM component and has been shown to be involved in the immune response to viral infection by binding chemokines that guide leukocyte extravasation. Versican also provides a barrier for leukocyte migration, as summarized in the contribution by McMahon et al. ADAMTS4 and ADAMTS5 are the two major versicanases (Santamaria et al., 2019), so it does not come as a surprise that they have been implicated in the pathological ECM remodeling that occurs upon viral infection. However, their role seems to be radically different. ADAMTS5 versicanase activity seems to exert a beneficial role by facilitating leucocyte migration as demonstrated by the delayed virus clearance observed in *Adamts5* knockout mice (McMahon et al., 2016). On the other hand, ADAMTS4 expression positively correlates with severe influenza virus infection in humans (Boyd et al., 2020), suggesting that ADAMTS4 can be a potential target for therapeutic intervention. Several inhibitory antibodies have been described (Santamaria and de Groot, 2019) and these can be deployed to target ADAMTS4 and other metzincins involved in the dysregulated immune response to viral infection. On the other hand, increasing the activity of ADAMTS5 in the context of severe influenza infection may not be trivial. Although increased ADAMTS5 activity has been achieved indirectly by blocking its endocytosis with a monoclonal antibody (Santamaria et al., 2017), a caveat when considering ADAMTSs as therapeutic targets is their complex, multisystemic role, for example their involvement in cardiovascular homeostasis (Santamaria and de Groot, 2020).

Like ADAMs, Astacins can cleave membrane-tethered proteins and therefore act as sheddases (Sterchi et al., 2008; Peters and Becker-Pauly, 2019). Meprin β is perhaps the best characterized Astacin. Meprin β is a type I transmembrane protein whose expression is associated with certain types of cancer, including melanoma. Thus, it is crucial to find the driver mutation(s) of meprin β and its variants to study the impact on the invasiveness of tumor cells. Two genetic variants of meprin β (G45R and G89R) have been found in melanomas. Gellrich et al. characterized these two variants and found that, similar to wild type meprin β, the G45R variant is able to shed a number of specific substrates and promote invasion when expressed in HeLa cancer cells. On the other hand, the G89R variant showed impaired trafficking to the cell surface, reduced shedding of its target substrates and diminished cancer cell invasion. A similar mechanistic approach can be extended to elucidate the activity of other meprin β variants (Peters and Becker-Pauly, 2019).

Overall, the studies included in this Research Topic expand our knowledge of the role and regulation of ADAMs, ADAMTSs and Astacins in a variety of biological processes, something that would not have been possible without the powerful new -Omics tools that have been widely adopted in the last decade.
